# Respectful closure of a CEnR DNA integrity study: A bridge to sustained interactions with research participants

**DOI:** 10.1017/cts.2025.82

**Published:** 2025-04-28

**Authors:** Martha I. Arrieta, L. Lynette Parker, Erica Sutherland, Robert W. Sobol

**Affiliations:** 1 Center for Healthy Communities, USA Health, University of South Alabama, Mobile, AL, USA; 2 USA Health Clinical Trials Office, Mobile, AL, USA; 3 Mitchell Cancer Institute, University of South Alabama, Mobile, AL, USA; 4 Department of Pharmacology, College of Medicine, University of South Alabama, Mobile, AL, USA; 5 Department of Pathology and Laboratory Medicine, Warren Alpert Medical School & Legorreta Cancer Center, Brown University, Providence, RI, USA

**Keywords:** Community engaged research, study closure, trust, sustained interaction, long-term connections

## Abstract

**Introduction:**

Community engagement in research (CEnR) is fundamental to recruitment and retention in research studies. CEnR study closure, with a view to promote subsequent interactions with participants, can foster long-term relationships between research teams and participants. We detail the principles, procedures and outcomes of respectful closure in a study focused on scaling-up tools to measure DNA integrity in population samples.

**Methods:**

The study incorporated CEnR principles and practices, engaging a Community Advisory Board (CAB) to guide most study procedures. The CAB-designed closure protocol included 1) attempts at one-on-one contact via telephone, followed by a letter, if no contact was established; 2) provision of a study closure packet; 3) periodic mailing of study updates; and 4) a request for sustained interaction with the Community Engagement Team (CE Team), including participants’ approval to receive invitations for future projects. Items 3 and 4 were framed as choices to further interaction and its extent.

**Results:**

Among 191 participants enrolled, 119 were contacted at closure (62% retention rate). Most frequently (97.5%), contacted participants agreed to receive information about new research projects, while 90.8% agreed to receive ongoing information about the DNA integrity study. Subsequently, the CE Team implemented two study update mailings and two CEnR studies, enrolling 18 participants in a consultative role and four in a collaborative role.

**Conclusions:**

Respectful study closure offers avenues for sustained interaction between CEnR teams and study participants, beyond the discrete boundaries of specific research projects. It can support the long-term connections that enable the positive outcomes of CEnR.

## Introduction

Community engagement in research (CEnR) embodies a continuum of community involvement in the research process, representing degrees of connection between researchers and members of the communities affected by the health issues addressed through studies. Because it generates the space whereby community interests, expertise, and real-life context are included to inform all stages of research, CEnR is considered a core element in the promotion of health equity and in the acceleration of translation [1–4].

A critical contribution of CEnR involves the promotion of participation and retention in the studies that advance scientific knowledge, specially as investigators approach communities whose members are traditionally underrepresented in biomedical research (UBR) [4–7]. Relevant CEnR strategies have been documented and apprised [8–12]. Moreover, the literature shows that long-term engagement with communities via durable partnerships with community organizations, leaders, and community members fosters trust in academic investigators and the research process, trust being fundamental to realize the full impact of CEnR [8–12].

Community Advisory Boards (CABs) hold a significant place in CEnR. They typically involve members of the communities whose residents will be asked to participate in research. Because CAB members are trusted in their communities, they lend a measure of credibility to researchers and their studies and provide access to interaction with potential study participants through their networks. Additionally, they effectively contribute to the development of tailored recruitment materials and study processes. CABs are fundamental to help research teams address the needs and concerns of participants in genomic research [13], where issues of mistrust, including fear of misuse of genetic information and of potential genetic discrimination, may prevent enrollment in studies [13–16].

Besides considerations of effective recruitment and retention, a fundamental goal of CEnR entails long-term commitment to the community and participants beyond the duration of any particular study [2,3,5,11,12]. In this context, the closure of a CEnR study generates another opportunity to strengthen the connection with study participants and foster valuable long-term relationships between community members and CEnR teams [2,3].

Translational research is an interdisciplinary endeavor, particularly when bridging the gap between basic and population-based research [17], such as the integration into population studies of new technologies in the field of DNA damage and repair [18]. We have contributed to this field via a CEnR study focused on scaling-up tools to measure DNA integrity in population samples [19,20]. The five-year study, a partnership between a genomic science lab and a community engaged health equity research center (CE Team), enrolled study participants from UBR populations in a mid-sized city in the US deep South [19,20]. A CAB guided all aspects of participant recruitment, enrollment, and retention [20]. We completed desired enrollment earlier than anticipated, requiring study closure by the end of year four. The CAB and CE Team designed the study closure protocol with the intention of fostering long-term relationships with study participants. Here we describe the CEnR features of the study and highlight elements of the closure process that promoted sustained interaction with participants. The recruitment and implementation of the CAB as well as all procedures for participant recruitment, data collection, and study closure were reviewed and approved by the Institutional Review Board of the University of South Alabama.

## Materials and methods

### The DNA integrity study

This longitudinal study proposed to quantify genome damage and DNA repair capacity in peripheral blood mononuclear cells isolated from blood samples provided by community dwelling individuals at two-year intervals. It proposed to enroll participants among residents of 11 zip codes, a source population (*n* = 115,633) where 69.4% of community members are of African American descent [27]. In partnership with the CAB, the CE team was responsible for implementing community informed recruitment, data collection, and periodic retention interactions. The lab performed all DNA integrity measures [19,20].

#### Study sample

Based on lab processes considerations, the study sample was to include 240 participants providing data every two years. The CE team used snowball sampling methodology for recruitment [28]. Members of a research cohort developed in a previous community health study were invited to participate. Enrolled participants had the option to refer friends or relatives, who in turn made additional referrals. Because persons referred often lived in zip codes other than the 11 defining the source population, the study area was expanded to eventually include 20 zip codes.

### CE team

By the study start, the center (which is located in the study area) had a 15-year history of community engaged work with partners in the source population [21–26]. It had implemented two community-led projects, and it had involved a CAB as well as employed community members as research apprentices in a third study [21,23,24,29]; the latter included the cohort of local residents which we further invited to participate in the DNA Integrity study.

The three-member CE Team was diverse, of Hispanic, Caucasian, and African American ancestry. Its leadership (MIA) and senior position (LLP) remained unchanged throughout study implementation. During the majority of the last two years of data collection and study closure, the third member was an experienced clinical trials assistant (ES). All CE Team members were well versed in CEnR principles and practices.

### CAB description

The CAB included eight women and two men of African American descent, all stakeholders within the communities comprising the source population. They had collaborated previously with the CE Team, five as community health advocates [25], two as research apprentices [24], and three as leaders of partner organizations [24,29]. They already had CEnR expertise and familiarity with the CE Team. The CAB periodically met with the CE Team to provide guidance regarding most study procedures, with each member receiving a $100 incentive to participation per meeting.

We have described elsewhere the processes and outcomes of the initial work by the CAB and CE Team [20]. In brief, over the course of thirteen meetings, this partnership resulted in the co-creation of : 1) a lay title for the study -from “Measuring genomic DNA damage and DNA repair capacity in longitudinal population samples – a step towards precision prevention” to “DNA Healing and Disease Prevention,” 2) an informational booklet explaining the study purpose and procedures, 3) a booklet explaining the rights of persons participating in research as well as the specific risks and benefits of participation in the study, and 4) the informed consent document and teach back questions to be used during the consent process. Additionally, the CAB carefully re-designed the processes and materials to be used to invite participants, advised on the approach to recruitment home visits, revised phone scripts for retention, and edited the letters to be mailed to participants not reached through retention phone calls.

Throughout their work, the CAB centered the principles of respect, agency, and confidentiality. They ensured the language used respected the shared history and experiences of the African American Communities they represented. Before initiating recruitment, the CAB led the CE Team to appraise elected officials representing the source population via a high-level meeting with investigators and university officials, in order to convey respect for the community as a whole [20]. In meetings throughout study years 2–5, the CAB consistently advised the CE Team at critical junctures, e.g., study re-initiation after halted recruitment due to COVID-19 and at the early study closure.

### Study procedures

The steps to contact former cohort members included: 1) a postcard invitation, 2) up to three phone calls, and 3) a letter reinstating the invitation. Both the postcard and the letter asked the potential participant to call a specific CE Team member if interested in learning more about the study. They included a mention of the monetary incentive to participation, but not the amount. Once the person called the CE Team, they were invited to participate in a face-to-face informational session explaining the study, with a choice of having the session occur in their home or at the center.

#### Recruitment

During the informational session, the CE Team used the informational booklet to explain the study purpose, longitudinal nature, procedures, the need to draw a blood sample, and what would be done with it. The CE Team mentioned the $125 incentive to participation and the opportunity to refer friends and relatives. Throughout the session, ample opportunity for questions and explanations was provided.

#### Enrollment

Upon completing the informational session, the CE Team queried the potential study participant’s interest in enrolling in the study. If the response was affirmative, the CE Team started the informed consent process, using the “Research Participant Rights” booklet to help guide the conversation and facilitate the review of the informed consent document. This included two instances for teach back dialog.

After signing the informed consent, a person was scheduled for a data collection visit at the center, followed by a visit to a nearby university laboratory where the blood sample would be taken, after which the cash incentive was disbursed. The incentive represented an acknowledgement of the time and resources (e.g., transportation) invested by the participants through the informational session, informed consent conversation, data collection visit, and visit to the laboratory for the blood sample draw.

For referred individuals, the CE Team initiated contact by calling the person at the number provided by the participant. If contact was established in the course of up to three phone calls, the invitation was made for an informational session at the person’s home or at the center. If no contact was made and an address had been provided, a letter was mailed asking that the person call the CE Team for more information. Once contact was established, the recruitment and enrollment procedures were the same as described above.

#### Data collection

Via in person interview, study participants provided self-reported basic health information, including sociodemographic data, history of chronic diseases, health insurance status, approximate monthly medical expenses, access to healthcare, preventive health screenings, and brief information about their food intake, smoking, and exercise habits. Anthropometric measurements (height, weight, waist and hip circumference) were taken. Finally, a 30 ml peripheral blood sample was obtained by a certified licensed practical nurse through venipuncture. Baseline and follow-up visits followed the same format.

#### Retention procedures

The CE Team called study participants at six-month intervals. The scripted conversation started with expressions of gratitude for their participation and continued enrollment, followed by a reminder that participation was voluntary, and a mention of their subsequent phone call and approximate date of their follow-up visit. We also offered to mail a short newsletter with study updates if the participant so desired. In the event of no contact, proxy relatives or friends (designated by the participant at enrollment) were called to learn of the person’s whereabouts. If phone contact was unsuccessful, a letter was mailed to the address on file asking the participant to call the center.

#### COVID-19 modifications

We had enrolled 21 participants when pandemic restrictions started. Only six (28.6%) had requested at home informational sessions. After a six-month recruitment hiatus, we discontinued home visits to minimize the risk of infections and asked potential participants to attend the center with strict adherence to COVID safety protocols. We made provisions for the blood draw to also occur at the center.

### CAB designed study closure procedures

Close to the end of study year four, the lab advised the CE Team that study objectives could be achieved with 80% of the intended study sample, and that follow-up blood samples were no longer necessary. Therefore, enrollment and data collection were stopped. The CE Team approached the CAB to explain the early study closure and ask how to proceed so that research participant contributions were honored.

Acting on the principles of respect and agency, the CAB developed a closure protocol comprised of: 1) attempts at one-on-one contact via telephone, followed by a letter if no contact was established; 2) the provision of a study closure packet; 3) periodic mailing of study updates through study year five and beyond; and 4) a respectful request for sustained interaction with the CE Team, including consent to receive invitations for future research projects.

Personal contact via phone was deemed appropriate as it had been the mainstay of interactions throughout retention procedures. The CAB scripted the conversation to consist of expressions of gratitude for study participation; an explanation of findings to date; assurance that analysis of the blood samples and other data collected continued; and a question about their permission and preferences regarding one or more levels of future contact. The choices offered were 1) receipt of a certificate of participation; 2) ongoing communication about study progress, status of analysis, and eventual study results; and 3) receipt of invitations to participate in future studies. Participants could choose among these options to create the relationship they wanted with the DNA Integrity study and the CE Team. Participants agreeing to options 2, 3, or both, which allowed for continued interaction between the participant and the CE Team, became part of the “sustained interaction group” (SIG).

The letter for persons not contacted by phone included the same information as the telephone script, and asked the study participant to call the CE Team with any questions and to convey their choice regarding sustained communication.

The closure package, construed to offer another layer of transparency by reiterating the reasons study recruitment and data collection had been stopped, was also meant to unambiguously convey the CE Team’s gratitude to participants for their critical role in the research, while providing assurance that ongoing communication about the study progress and eventual results would be forthcoming. It included: 1) a letter explaining the study had closed to new recruitment and to follow up procedures, 2) a copy of the first published article [19], 3) a plain language version of the article’s abstract, 4) a Season’s Greetings card, and, except for those who had declined to receive it, and 5) a certificate of appreciation for their participation in the study.

## Results

We contacted 51 (45.5%) of the 112 participants retained at the end of the previous cohort study. Nineteen of those decided to participate in the DNA Integrity study and provided an average of two referrals. By the study’s early closure, the CE Team had recruited 191 community members, 90% (*n* = 172) through referrals. Thirty-four participants had a follow up visit. During closure interactions, we were able to contact 119 participants (62% overall retention rate, 35% loss to follow up, 2% censored, and 1% withdrawn).

The demographic composition of the study sample at baseline and at closure, as well as the rate of loss to follow-up by demographic subcategories, are detailed in Table 1. Attrition rates were higher among the youngest age group, males, those with lower levels of education, unemployed participants, those with the lowest level of monthly income, and persons who were not of African American descent.

**Table 1.** Demographic composition of study sample at baseline and closure, rates of loss to follow up by demographic sub-categories

Characteristic	Baseline			Closure completed			Loss to follow up*	
	No.	%		No.	%		No.	%**
Age (years)								
18–44	63	33.0		30	25.2		33	52.4
45–64	91	27.6		62	52.1		29	31.9
65 and over	37	19.4		27	22.7		10	27.0
Sex								
Female	119	62.3		78	65.6		41	34.5
Male	72	37.7		41	34.4		31	43.1
Race								
African American	184	96.3		119	100		65	35.3
Other***	7	3.7		_	_		7	100.0
Education								
Less than high school	35	18.3		18	15.1		17	48.6
High school diploma or GED	76	39.8		40	33.6		36	47.4
Vocational degree	18	9.4		16	13.5		2	11.1
Associate’s degree or some college	51	26.7		39	32.8		12	23.5
Bachelor’s degree	11	5.8		6	5.0		5	45.5
Employment								
Working full- or part-time	56	29.3		33	27.7		23	41.1
Unemployed	47	24.6		26	21.8		21	44.7
Disabled	49	25.7		34	28.6		15	30.6
Retired	30	15.7		19	16.0		11	36.7
Homemaker or student	9	4.7		7	5.9		2	22.2
Monthly income								
$0–$499	29	15.2		14	11.8		15	51.7
$500–$999	57	29.8		38	31.9		19	33.3
$1,000–$1,499	46	24.1		28	23.5		18	39.1
$1,500 or greater	52	27.2		34	28.6		18	34.6
Not sure or Missing	7	3.7		5	4.2		2	28.6

* Four censored (2 moved out of state, 2 deceased) and two withdrawn

** Within demographic sub-categories

*** Two Caucasian, four mixed race, one ambiguous answer

Study closure per protocol was completed for 103 participants over two and a half months. Overlapping eight weeks with the closure period, we contacted 29 participants with invitations to join focus groups exploring COVID-19 knowledge and perceptions. Such contacts doubled up as closure interactions for 11 participants. Additionally, five individuals called the CE Team, four to inquire about the focus group study and one to ask about timing of a follow-up visit. Those self-initiated interactions were used by the CE Team to provide study closure information. Closure interactions were done exclusively via phone for 95% of participants and 5% by participants’ phone response to mailed letters.

### Agreement to sustained interaction

We attained complete information regarding sustained interaction for 108 (90.7%) of the 119 participants contacted at study closure. For nine others (7.6%), we only recorded information about agreement to be contacted for new research studies. Two study participants (1.7%) had missing information for all the sustained interaction questions.

Regarding the three options for sustained interaction, participants most frequently (*n* = 116, 97.5%) agreed to be contacted with information about new research studies. This was followed by agreement to receive ongoing information about the DNA integrity study (*n* = 108, 90.8%). Three individuals declined receipt of a certificate of participation (2.5%). Overall, 105 persons agreed to all the continued communication options, while 117 (98.3%) agreed to one or both of the options that defined membership in the SIG.

### Sustained interaction activities

After study closure, over the course of fifteen months, the CE Team implemented two study update mailings (at six-month intervals) and conducted two other CEnR studies, recruiting and enrolling participants among eligible members of the SIG.

#### Study updates

The first mailing included a letter by the principal investigator (RWS), explaining his move to a different university and the work towards reconfiguring the lab at the new location, representing a temporary lapse in the analysis of the blood samples. The packet also contained information about a poster presentation by the CE Team at the Translational Science Conference held in Washington DC in April 2023 [30], alongside the published abstract and its plain language version.

The second mailing comprised an introductory letter by the CE Team and a pictorial report describing the newly conformed Genomic Lab Team and the status of re-initiated work on sample analysis, a copy of the second article published [20], a plain language summary of the article’s abstract, and a Season’s Greetings card. This mailing was implemented past the end of study year five, which marked the formal end of funding for the community engagement component of the project. Both the CE Team and the Genomic Lab Team are committed to providing updates to those participants who requested them, inclusive of both preliminary and final study results as they become available over the next few years.

#### Invitations for new CEnR studies

The first study involved a consultative role, the tailoring of essential COVID-19 messages for an informational campaign focused on COVID-19 literacy. Among 41 eligible SIG members, 33 (80.5%) were contacted with an invitation to participate in one of five focus groups to discuss the value, relevance, and wording of a set of elemental COVID-19 messages. Twenty-four of those contacted accepted the invitation and 18 attended their corresponding focus group (43.9% response rate). Those who declined (*n* = 8), and one person responding after recruitment was complete, still expressed interest in continued information about new research studies.

The second study sought to engage community members in a collaborative role for the co-creation of informational materials regarding medical research, to be used in community-based sessions promoting understanding and familiarity with the research process. Nine persons among the SIG were eligible to constitute a “Research Partner Committee” scheduled to meet seven times over five weeks to develop materials for two informational sessions related to fundamental concepts of medical research and the protections in place for persons participating in studies. The meetings were planned to last two and a half hours each. Among the seven persons contacted, four accepted the invitation and participated in seven co-creation meetings (44.4% participation rate). The other three were not able to participate due to the intensive schedule and duration of the meetings.

Overall, the invitations we extended involved 41 SIG members, nine of them meeting eligibility criteria for the two studies. In the end, 18 (43.9%) of these SIG members participated in the evaluation of COVID health literacy messages, resulting in a community informed COVID literacy campaign. Four of nine eligible SIG members also participated in a collaborative role, resulting in the co-creation of informational materials regarding medical research.

## Discussion

The interdisciplinary partnership between a genomic science lab and a mature CEnR team resulted in effective implementation of a study aiming to accelerate population-based applications of technologies to quantify DNA damage and repair. We enrolled 191 UBR participants in a DNA integrity study with 62% retention over three years. Through guidance and oversight by the CAB, respectful engagement principles and attitudes permeated all study interactions [20], and recognized CEnR practices were implemented to foster UBR participant recruitment and retention [8–12]. The process and outcomes of closure procedures, pursuant to promoting a long-term relationship between study participants and the CE Team, resulted in agreement to at least one of two possible choices for sustained interaction by 117 (98.3%) of participants contacted at the study’s early closure. Subsequently, the CE Team involved 18 of them in a consultative role, and four in a collaborative role, while implementing new CEnR studies.

### CEnR framework for the study

The center’s substantive history of engagement with study area stakeholders and their communities allowed the CE Team to consult a robust CAB, capable of effectively addressing the challenges of the DNA integrity study by organically recommending principles and practices that are proven CEnR staples [8–12]. Also, the CE Team’s commitment to and expertise in community engagement contributed to principled and efficient implementation of CAB recommendations, where the value placed on forging respectful relationships with study participants permeated all interactions. For example, aligned with the practice of ensuring comprehension and transparency, both an informational and a rights of research participants booklet were co-created [8–10,12,20]; a monetary incentive to participation that recognized the investment of time and resources by study participants was offered [8,10,12]; and the language used for scripted interactions was respectful of the participants’ shared history and experiences [9–11].

### Value, principles and practices of respectful study closure

Both the CAB and CE Team recognize that long-standing partnerships with research participants, and by extension, their communities, are a hallmark of CEnR [10–12]. Thus, their commitment to craft closure procedures respectful to participants while also promoting long-term relationships with the research team. Respectful study closure allows for recognition of partners’ and participants’ contributions to the study [31]. It fosters sustained bidirectional communication through timely interactions about the particulars of study closing, while listening to concerns and responding to questions [31–33]. Moreover, through the sharing of preliminary results and/or establishing the timelines for direct communication of study progress and findings, investigators demonstrate their regard for partners and participants, acknowledge the importance of their contributions, and further the community members’ understanding of what research is and how the results are used [34–36].

#### Principles: recognition, respect, and agency

While discussing the early study closure, the CAB and the CE team agreed that closure interactions should: 1) honor the contribution of study participants to the furthering of DNA integrity measurement science, 2) promote a long-term relationship with the CE team and the health equity center, and 3) foster goodwill towards medical research in general. Accordingly, a certificate of participation in the research study was created to convey the importance of the participants’ contribution to the advancement of DNA Integrity measurement science and represent an acknowledgment of the fundamental role of participants in the generation of knowledge, as discussed by Fernandez, Kodish and Weijer [33].

Another guiding principle in CAB recommendations was “agency,” i.e., the right of participants to choose whether they wanted continued interaction with the CE Team and its academic institution, and if so, the level of interaction they preferred. Integral to the respect due to study participants, the CAB stressed the importance of asking permission to engage them in any of the proposed sustained interaction steps [33,36]. In the present study, the rate of positive responses to sustained interaction questions was quite high, but not uniform or universal, representing the exercise of individual decision making by study participants.

#### Practices

*Personal interaction:* The CAB’s recommendation that the initial closure interaction be conducted on an individual basis over the phone aligns with the “Participant FIRST” guidelines defined for the closure of AD clinical trials [31,37]. One-on-one interaction facilitates dialogue specific to the interest, concerns and circumstances of each study participant. In our case, the closure phone calls and any related communications were implemented by the two members of the CE Team who had been responsible for the vast majority of recruitment and data collection activities. In this way, closure procedures were a seamless new step in the participants’ experience, capitalizing on the familiarity forged through routine retention encounters [31,33,37].

*Periodic mailing of study updates:* Another CAB recommendation, also supported by the literature [31–33,26], involved the provision of ongoing written communications with specific components tailored to the choices made by participants at study closure. At the time of this writing, we have provided the SIG two study updates via mail. We have chosen to be inclusive rather than scarce in the materials shared, considering that each person will make a choice regarding what to pay attention to. As recommended in the literature [33,35], we have provided lay language versions of the abstracts corresponding to the two articles and poster presentation, offering a context for any material enclosed via relevant introductory letters also written in plain language. Even though there are low literacy concerns for UBR populations, a broad approach to sharing information about study progress and results has been a preference voiced in studies of participants’ perceptions of their involvement in research, as well as of researchers who have explored the matter [33–36,38].

*Direct dissemination of study information:* The conversation about study closure included the offer of periodic study updates with information on the progress of analysis of the blood samples participants had contributed, as well as both partial and final study results. The option of receiving periodic updates honored the right of study participants to know how the data and samples they provided were being used within the scientific endeavor, and, eventually, the ultimate results of such endeavor [33,34,36]. Implicit in informing participants of the progress and results of a study is the recognition that the person providing the data, not just the data itself, is central to the research process [35,39].

*Promotion of sustained interaction:* As stressed by the CAB, study closure offered an opportunity to establish channels for sustained interaction beyond the immediate objectives of the DNA integrity study. The CE Team’s commitment to provide periodic updates on the study progress created a space for ongoing interaction with participants, as well as opportunities to capitalize on the rapport established during data collection encounters and offer a wider view of the ways the academically-based research center pursued its fundamental CEnR goal. Moreover, in asking study participants whether they would like to be appraised of new research opportunities, the CE Team created an opening to foster a bi-directional dialog that allowed deeper understanding of the study participants’ values and circumstances, as they accepted or declined participation in successive studies. Ongoing interaction holds the promise of a stronger connection with those community members who agreed to continued communication with the CE Team.

### Study strengths

Seminal to the success of closure procedures was the commitment of the DNA Integrity study PI, a highly specialized basic science investigator, as well as of the CE Team and institution, to honor fundamental principles of CEnR through its interactions with the CAB, community stakeholders, and study participants [20]. This was evident in the provision of project-specific financial resources to support sustained engagement interactions throughout the fifth year of the grant, even as the study PI and Lab migrated to a new academic institution. Once direct funding lapsed, the health equity center underwrote subsequent work by the CE Team to implement sustained interaction activities. Going forward, the study PI, the CE team, and the center are committed to the implementation of ongoing updates and communication of the final study results.

Although the investment of time and resources in support of respectful study closure procedures and attendant ongoing communication of results have been mentioned as challenges to what is, by definition, a long-term commitment in CEnR studies [2,4,40], there is a growing recognition of the need to factor in the requisite resources in time and budget when CEnR projects are first designed, as well as for funders to offer mechanisms that can support sustained community engagement beyond the limits of discrete project endeavors [1,31,32].

A second strength of the study was its longitudinal nature, whereby routine cohort retention procedures allowed for iterative contact with study participants. When the study ended earlier than expected, there was already a sense of comfort on both sides of the interaction which facilitated the implementation of respectful closure procedures, inclusive of ongoing communication if so elected. This was in contrast to situations where abrupt cessation of interactions is perceived by participants as a one-sided behavior that disappoints and devalues their contribution [34].

Also favorable to the sustained interaction outcomes we report was the expertise and stable makeup of the CE Team over the formal course of the study and beyond. This allowed for consistent interaction within an environment that welcomed and honored participants as collaborators in the research.

### Study limitations

We acknowledge two limitations of the study: sample attrition (35%) and incomplete data on the sustained interaction variables for 9.2% of the participants contacted at study closure.

In terms of attrition, engaging and retaining UBR participants in clinical studies is recognized as a challenge, also operating in the DNA Integrity study despite its CEnR nature. Undoubtedly, successful recruitment was facilitated by the initial accrual method inviting persons who had a prior experience of interaction with the CE Team. However, the vast majority of persons enrolled through referrals were new in their relationship with the CE Team and the Center. As such, recognized barriers to retention, from the practical (changing life circumstances, lack of interest in the research) to the fundamental (mistrust and suspicion), were likely at play among newly engaged study participants [1,7,9].

In terms of incomplete data on sustained interaction, we acknowledge that overlap between recruitment for a concomitant study and closure procedures resulted in instances where the closure protocol was not strictly followed. Thus, the instances of missing data are due to a procedure failure by the CE Team, rather than to lack of response by study participants.

## Conclusions

Within our experience of the respectful closure of a CEnR study, we were able to foster sustained interactions beyond study closure with a vast majority of retained participants. The ongoing interactions, related to the provision of study updates, the sharing of intermediate research outcomes, and invitations to participate in new studies have opened a space to consolidate and expand the research team’s understanding of community member’s values, interests, and circumstances. They have also facilitated a deeper understanding by community members of the goals, work, and *modus operandi* of the engaged research team, and by extension, of the academia-based center. The opportunity for sustained interaction brought forward by a respectful study closure holds great potential to foster the beneficial outcomes of CEnR in terms of long-term, trust-generating, bi-directional relationships with community members who have participated in research.
